# MAXWELL A. POLLACK AWARD FOR CONTRIBUTIONS TO HEALTHY AGING PRESENTATION AND LECTURE

**DOI:** 10.1093/geroni/igae098.0668

**Published:** 2024-12-31

**Authors:** Debra Dobbs

**Affiliations:** Chair

## Abstract

The Maxwell A. Pollack Award for Contributions to Healthy Aging Lecture will feature an address by the 2023 Pollack Award recipient Bei Wu, PhD, FGSA, FAGHE of New York University. This session will also include the presentation of the 2024 Maxwell A. Pollack Award to recipient Edward Alan Miller, PhD, MPA, FGSA, of the University of Massachusetts Boston. The Maxwell A. Pollack Award for Contributions to Healthy Aging Award recognizes instances of practice informed by research and analysis, research that has directly improved policy or practice, and distinction in bridging the worlds of research and practice.

